# Is the International Normalized Ratio (INR) Enough? Reevaluating INR-Based Decision-Making for Four-Factor Prothrombin Complex Concentrate in Geriatric Warfarin-Associated Intracranial Hemorrhage: A Multicenter Cohort Study

**DOI:** 10.3390/biomedicines14102190

**Published:** 2026-09-28

**Authors:** Yunus Dogan, Akkan Avci, Ramazan Guven, Ozgur Sogut, Serkan Dogan, Mehmet Akif Karamercan, Ertugrul Altınbilek, Ertugrul Altug, Begum Seyda Avci, Adem Az, Gokhan Eyupoglu, Said Kural, Ogulcan Alp, Metin Ozmen, Ozgur Guven, Yeliz Simsek, Erdem Aksay, Ahmet Burak Urfalioglu, Pelin Oguz Tura, Hakan Kaan Yilmaz, Sara Tunca, Can Ilcin, Efe Demir Bala, Munisahan Guvercin

**Affiliations:** 1 Department of Emergency Medicine, Haseki Research and Training Hospital, University of Health Sciences, Istanbul 34096, Turkey; yunusdogan1734@gmail.com (Y.D.); ozgur.sogut@sbu.edu.tr (O.S.); adem.aaz@gmail.com (A.A.); 2 Department of Emergency Medicine, Adana City Research and Training Hospital, University of Health Sciences, Adana 01230, Turkey; akkan.avci@atlas.edu.tr (A.A.); begumtngnr@hotmail.com (B.S.A.); ylzberk@yahoo.com (Y.S.); erdemaksay@gmail.com (E.A.); aburakurfali969@hotmail.com (A.B.U.); pelinoguzz@gmail.com (P.O.T.); 3 Department of Emergency Medicine, Faculty of Medicine, Atlas University Hospital, Istanbul 34408, Turkey; 4 Department of Emergency Medicine, Kanuni Sultan Suleyman Research and Training Hospital, University of Health Sciences, Istanbul 34303, Turkey; serkandogan1303@gmail.com (S.D.); canilcin@gmail.com (C.I.); edbala97@gmail.com (E.D.B.); munisahanguvercin@gmail.com (M.G.); 5 Department of Emergency Medicine, Gazi University Hospital, Faculty of Medicine, Gazi University, Ankara 06500, Turkey; makaramercan@yahoo.com (M.A.K.); dr.ozgurguvenn@gmail.com (O.G.); 6 Department of Emergency Medicine, Hamidiye Sisli Etfal Research and Training Hospital, University of Health Sciences, Istanbul 34485, Turkey; ertugrulaltinbilek@gmail.com; 7 Department of Emergency Medicine, Basaksehir Cam and Sakura City Hospital, University of Health Sciences, Istanbul 34480, Turkey; ertugrulaltug42@gmail.com (E.A.); gokhanbey982@gmail.com (G.E.); saidkural@gmail.com (S.K.); ogulcanalpmd@gmail.com (O.A.); drmetin@outlook.com (M.O.);yilmazhakankaan@gmail.com (H.K.Y.); saratunca@hotmail.com (S.T.)

**Keywords:** four-factor prothrombin complex concentrate, international normalized ratio, anticoagulation reversal, geriatric, mortality

## Abstract

**Background/Objectives:** Warfarin-associated intracranial hemorrhage (WA-ICH) remains one of the most devastating anticoagulant-related emergencies, particularly among older adults, with substantial morbidity and mortality. Although current guidelines recommend that reversal strategies should be guided primarily by bleeding severity and clinical presentation rather than the international normalized ratio (INR) alone, real-world treatment decisions often continue to rely on INR thresholds. This study investigated whether INR is an adequate determinant for four-factor prothrombin complex concentrate (4F-PCC) administration in geriatric patients with non-traumatic WA-ICH. **Methods:** In this multicenter retrospective cohort study, consecutive patients aged ≥65 years presenting with non-traumatic WA-ICH to five tertiary-care centers in Turkey were evaluated. Patients were categorized according to reversal strategy into a 4F-PCC group (*n* = 30) and a heterogeneous non-PCC management group (*n* = 30), which included supportive care, intravenous vitamin K, FFP, combinations of these approaches, or no specific reversal treatment. Subgroup analyses were performed using multiple INR thresholds to examine the relationship between INR levels, treatment allocation, and in-hospital mortality. The primary outcome was all-cause in-hospital mortality. **Results:** Patients who received 4F-PCC had higher admission INR values than those managed without PCC, whereas admission Glasgow Coma Scale (GCS) was similar between treatment groups. Admission GCS was substantially lower among non-survivors than survivors. In the revised Firth-penalized logistic regression model, higher admission GCS was independently associated with lower odds of in-hospital mortality (aOR 0.37 per 1-point increase; 95% CI 0.10–0.62; *p* < 0.001), whereas 4F-PCC administration and admission INR values were not independently associated with mortality. **Conclusions:** Higher admission INR was associated with 4F-PCC administration and was not independently associated with mortality, whereas admission GCS was independently associated with in-hospital mortality. These findings support consideration of neurological, radiological, and laboratory information together rather than interpretation of admission INR in isolation. Given the retrospective design, small sample size, baseline differences in anticoagulation intensity, and potential residual confounding, the findings should be considered hypothesis-generating.

## 1. Introduction

Warfarin continues to play a substantial role in the prevention and treatment of thromboembolic disorders, including atrial fibrillation, venous thromboembolism, and thrombosis associated with prosthetic heart valves. Although the adoption of direct oral anticoagulants (DOACs) has reduced the overall use of vitamin K antagonists (VKAs) in many healthcare systems, warfarin remains indispensable in specific patient populations and continues to be widely prescribed in several countries, including Turkey, where it is the principal VKA available in routine clinical practice [1,2,3,4]. Consequently, warfarin-associated bleeding complications remain an important clinical challenge, particularly in aging populations with a high burden of cardiovascular comorbidities.

Among the complications associated with warfarin therapy, intracranial hemorrhage (ICH) is the most devastating and is associated with substantial mortality, severe neurological disability, and prolonged healthcare utilization [5]. The incidence and clinical consequences of warfarin-associated ICH (WA-ICH) are particularly pronounced in older adults owing to increased frailty, multimorbidity, cerebral small-vessel disease, and age-related vulnerability to hemorrhagic injury [5,6]. Anticoagulant-associated ICH is characterized by larger hematoma volumes, increased risk of hematoma expansion, and poorer functional outcomes compared to non-anticoagulant-related hemorrhage, emphasizing the need for rapid and effective anticoagulation reversal [6,7].

Prompt reversal of anticoagulation is a cornerstone of WA-ICH management. Current international guidelines, including those issued by the American Heart Association/American Stroke Association (AHA/ASA) and the American College of Cardiology (ACC), strongly recommend the administration of four-factor prothrombin complex concentrate (4F-PCC) in combination with intravenous vitamin K for patients presenting with life-threatening VKA-associated bleeding [8,9,10,11,12,13]. Compared to fresh frozen plasma (FFP), 4F-PCC provides more rapid restoration of vitamin K-dependent clotting factors, faster normalization of the international normalized ratio (INR), lower infusion volumes, and reduced treatment delays, which are factors that may contribute to limiting ongoing bleeding and hematoma expansion [8,9,10,11,12,13,14]. In Turkey, 4F-PCC is administered according to weight- and INR-based dosing recommendations to achieve individualized anticoagulation reversal [9,10,11].

However, important uncertainties remain regarding patient selection and treatment decision-making in terms of 4F-PCC use. In routine clinical practice, INR frequently serves as a major determinant of reversal intensity, and in some settings, influences the decision to administer PCC. However, INR is primarily a laboratory measure of anticoagulation intensity and may not adequately reflect the biological complexity of ICH severity, hematoma dynamics, neurological impairment, or overall prognosis [2,15]. Increasing evidence suggests that factors such as hemorrhage location, hematoma volume, level of consciousness, frailty status, and underlying comorbidities may influence clinical outcomes more than INR values alone [15,16,17,18].

Importantly, although 4F-PCC consistently achieves rapid correction of warfarin-induced coagulopathy, whether this laboratory improvement translates into better patient-centered outcomes remains uncertain. Most studies have focused on INR normalization as a surrogate endpoint, whereas clinically relevant outcomes such as mortality, neurological recovery, and functional status have been investigated less extensively [14,15,16,17,18]. Consequently, INR remains deeply embedded in treatment algorithms despite limited evidence demonstrating that it independently predicts clinical benefits from reversal therapy.

Moreover, the widely held assumption that patients with higher INR levels derive greater benefit from PCC administration has not been adequately validated. Studies have reported conflicting results regarding the association between admission INR, hematoma expansion, and mortality, and only a limited number have evaluated whether INR modifies the effectiveness of PCC treatment itself [2,15,16,17,18,19]. Accordingly, the clinical implications of INR-based treatment thresholds remain uncertain, particularly when laboratory anticoagulation intensity is considered without the broader clinical and radiological context.

This uncertainty is particularly relevant in geriatric populations. Older adults account for a substantial proportion of WA-ICH cases and experience disproportionately high mortality and disability; however, they remain underrepresented in studies that assess the clinical utility of PCC and the prognostic significance of INR [5,18,19,20]. Furthermore, few studies have used real-world multicenter data to examine whether admission INR meaningfully influences survival among older patients receiving PCC. Hence, it remains unclear whether INR should be viewed primarily as a dosing parameter or as a treatment-selection criterion in the management of geriatric WA-ICH.

In this study, we evaluated whether admission INR meaningfully influences the relationship between 4F-PCC administration and survival in older adults with non-traumatic WA-ICH. Rather than examining INR solely as a marker of anticoagulation intensity, we assessed whether INR-based treatment decision-making reflects clinically relevant outcome differences.

## 2. Materials and Methods

### 2.1. Study Design and Setting

This multicenter retrospective observational cohort study was conducted across five tertiary-care hospitals in Turkey. Consecutive eligible patients presenting to the emergency department (ED) with non-traumatic ICH while receiving warfarin therapy were identified through institutional electronic health record systems. All eligible patients identified during the prespecified study period were included consecutively. No matching, quota sampling, predefined group size, or fixed stop rule was applied according to treatment status.

Data were retrospectively collected between 15 June 2024 and 30 August 2024. All variables were extracted from fully anonymized electronic medical records. Investigators had no access to personally identifiable patient information during data acquisition, analysis, or reporting. The study was designed and reported in accordance with the Strengthening the Reporting of Observational Studies in Epidemiology (STROBE) recommendations for observational research and followed the “retrospectoscope” framework [21] to minimize methodological bias in retrospective emergency medicine studies.

The study protocol was approved by the Ethics Committee of Başakşehir Training and Research Hospital (Approval Code: E-963117027-514.10-248520966; 16 July 2024). Institutional permissions were obtained from all participating centers. Given the retrospective nature of the study and the use of anonymized data, the requirement for informed consent was waived by the ethics committee. The study was conducted in accordance with the principles of the Declaration of Helsinki.

### 2.2. Study Population

Patients aged 65 years or older presenting to the ED with non-traumatic WA-ICH were eligible for inclusion. WA-ICH was defined as radiologically confirmed spontaneous ICH in a patient with documented active warfarin use at the time of ED admission.

Eligible cases were identified using the International Classification of Diseases, Tenth Revision (ICD-10) diagnostic codes for non-traumatic ICH, including subarachnoid hemorrhage (I60), intracerebral hemorrhage (I61), and other non-traumatic intracranial hemorrhages (I62). The diagnosis was subsequently verified through review of neuroimaging findings and medication records.

Patients were included if they met all of the following criteria: age ≥ 65 years; active warfarin therapy at the time of presentation; radiologically confirmed non-traumatic ICH; availability of admission laboratory measurements, including INR; and availability of hospital outcome data. Patients were excluded if they had traumatic ICH; absence of documented warfarin use; missing essential clinical, laboratory, or radiological data; or inadequate follow-up information regarding in-hospital outcomes.

### 2.3. Anticoagulation Reversal Strategies

Patients were categorized according to the anticoagulation reversal strategy administered during ED management.

The 4F-PCC group included patients who received 4F-PCC (Cofact^®^; Prothya BioSolutions, Amsterdam, The Netherlands), irrespective of concomitant vitamin K administration. Because treatment decisions reflected routine clinical practice, documentation of intravenous vitamin K administration was collected when available but was not mandatory for classification within the 4F-PCC group.

The non-PCC group represented a real-world comparator cohort and included patients managed without PCC administration. Treatment in this group varied according to physician judgment and institutional practice and consisted of supportive management alone, intravenous vitamin K, FFP, a combination of these therapies, or no specific reversal treatment. 4F-PCC administration was guided by local institutional protocols based on body weight and admission INR. However, the exact center-specific dosing algorithms and patient-level administered doses were not consistently retrievable across all participating centers; therefore, a reliable center-by-center comparison of Cofact^®^ administration protocols could not be performed.

Management in the non-PCC group was not standardized across participating centers and reflected routine clinical practice. It could include supportive care alone, intravenous vitamin K, FFP, combinations of these approaches, or no specific reversal treatment. Accordingly, the non-PCC group represented a heterogeneous management cohort rather than a uniform alternative reversal strategy.

### 2.4. Data Collection and Variables

Clinical, laboratory, and radiological data were extracted from electronic medical records using a standardized data collection form. Demographic variables included age and sex. Clinical variables included comorbid conditions and baseline neurological status as assessed by the Glasgow Coma Scale (GCS) at emergency department presentation. Physiological parameters recorded at ED admission included systolic blood pressure, diastolic blood pressure, and heart rate.

Laboratory variables consisted of admission INR, prothrombin time (PT), and activated partial thromboplastin time (aPTT). Post-treatment INR was assessed within 15–30 min after completion of the initial anticoagulation reversal intervention. For exploratory analyses, admission INR values were categorized according to predefined clinically relevant thresholds to evaluate whether the relationship between PCC administration and clinical outcomes differed across varying degrees of anticoagulation intensity.

### 2.5. Neuroimaging Assessment

All patients underwent cranial computed tomography (CT) at presentation. Neuroimaging findings were reviewed using radiology reports and imaging documentation. Hemorrhage characteristics included hemorrhage subtype, anatomical location, presence of intraventricular extension, and hematoma volume. For intraparenchymal hemorrhages, hematoma volume was calculated using the validated ABC/2 method [22]. Hemorrhage progression was defined as any radiologically documented increase in hematoma size or the development of a new hemorrhagic extension in follow-up CT imaging during hospitalization. The timing of follow-up CT imaging was not standardized across participating centers and was determined by the treating physician according to clinical status and local institutional practice.

### 2.6. Outcomes

The primary outcome was all-cause in-hospital mortality. Secondary outcomes included admission hematoma volume, the presence of intraventricular hemorrhage, intensive care unit (ICU) admission, radiological hemorrhage progression, an association between 4F-PCC administration and survival, and the relationship between admission INR categories and clinical outcomes. A prespecified subgroup analysis was performed to determine whether the association between 4F-PCC administration and in-hospital mortality varied across different INR strata.

### 2.7. Statistical Analysis

Statistical analyses were performed using IBM SPSS Statistics for Windows, version 30.0 (IBM Corp., Armonk, NY, USA). The distribution of continuous variables was evaluated using visual (histograms and Q-Q plots) and analytical methods (Shapiro–Wilk test). Normally distributed variables were reported as mean ± standard deviation (SD), whereas non-normally distributed variables were expressed as median and interquartile range (IQR). Categorical variables were summarized as frequencies and percentages.

Comparisons between treatment groups were performed using the Mann–Whitney U test for non-normally distributed continuous variables. Categorical variables were compared using Pearson’s chi-square test or Fisher’s exact test, as appropriate. For analyses involving small expected cell frequencies, the Monte Carlo exact test was applied.

To explore the clinical utility of INR as a treatment-selection parameter, stratified subgroup analyses were conducted across predefined INR categories. The association between PCC administration and in-hospital mortality was examined within each INR stratum to evaluate whether anticoagulation intensity modified the relationship between reversal treatment and survival. Given the limited sample size and sparse cell counts at several INR thresholds, these threshold-based analyses were considered exploratory and hypothesis-generating. No adjustment for multiple comparisons was performed, and individual *p*-values should therefore be interpreted cautiously rather than as evidence of validated INR cutoffs.

To account for potential confounding in the association between 4F-PCC administration and in-hospital mortality, an additional Firth-penalized logistic regression model was fitted with all-cause in-hospital mortality as the dependent variable. The model included 4F-PCC administration, age, admission INR, admission hematoma volume, comorbidity count, and admission GCS. GCS was additionally incorporated in the revised analysis to account for baseline neurological severity. The multivariable analysis was performed using complete cases, and adjusted odds ratios (aORs) with 95% confidence intervals (CIs) were reported.

All statistical tests were two-tailed, and a *p*-value < 0.05 was considered statistically significant.

## 3. Results

### 3.1. Patient Characteristics

In total, 60 geriatric patients (≥65 years) with non-traumatic WA-ICH were included in the analysis. Of these, 36 (60.0%) were female and 24 (40.0%) were male. Patients were categorized according to ED management into a 4F-PCC group (*n* = 30) and a non-PCC group (*n* = 30).

Baseline demographic and clinical characteristics were generally comparable between the groups (Table 1). The mean age was 77.3 ± 8.5 years in the 4F-PCC group and 75.5 ± 13.2 years in the non-PCC group (*p* = 0.524). Likewise, sex distribution, comorbidity profiles, admission blood pressure measurements, and heart rate did not significantly differ between treatment groups. Admission neurological status, assessed using the GCS, was also similar between the groups (median [IQR], 12.5 [7.25–15] in the 4F-PCC group vs. 13 [6–15] in the non-PCC group; *p* = 0.880). In contrast, baseline coagulation parameters differed between the groups. Pretreatment PT was higher in the 4F-PCC group than in the non-PCC group (median [IQR], 51.95 [26.60–135.80] vs. 31.00 [15.30–51.25] s, *p* = 0.018). Similarly, pretreatment aPTT was higher among patients receiving 4F-PCC (55.35 [37.65–74.73] vs. 42.30 [30.40–53.63] s, *p* = 0.016). Admission INR was also higher in the 4F-PCC group (5.90 [2.90–11.00] vs. 3.15 [1.33–4.98], *p* = 0.005), indicating an association between higher admission INR and 4F-PCC administration.

Despite these baseline differences, post-treatment INR values were comparable between the two groups (*p* = 0.476). Furthermore, the magnitude of INR reduction achieved during hospitalization did not significantly differ between groups (*p* = 0.104).

### 3.2. Neuroimaging Findings and Clinical Outcomes

Radiological characteristics were broadly similar between the treatment groups (Table 1). Neither admission hematoma volume nor changes in hematoma size in follow-up CT significantly differed between patients who received 4F-PCC and those who did not. Similarly, ED management characteristics, including endotracheal intubation and FFP administration, did not significantly differ between the groups. Vitamin K was administered to 12 patients (40.0%) in the 4F-PCC group and 7 patients (23.3%) in the non-PCC group (*p* = 0.165), indicating that concomitant vitamin K administration was not universal in either treatment group.

Overall, 25 (41.7%) patients died during hospitalization, including 13 (43.3%) in the 4F-PCC group and 12 (40.0%) in the non-PCC group. In the unadjusted comparison, in-hospital mortality did not differ significantly between the groups (*p* = 0.952). No significant differences were identified in overall clinical disposition or hospital outcomes (Table 1).

### 3.3. Distribution of Treatment According to Admission INR Thresholds

To explore the role of INR in treatment allocation, patients were stratified according to predefined admission INR thresholds (Table 2).

The proportion of patients who received 4F-PCC progressively increased with higher INR values. At the INR threshold of 2, nearly all patients in the 4F-PCC group had INR values ≥ 2, whereas a substantially larger proportion of patients in the non-PCC group had INR values < 2 (96.7% vs. 63.3%, *p* = 0.001). Similar differences were observed at increasingly higher INR thresholds, including INR ≥ 5 (56.7% vs. 26.7%, *p* = 0.018), INR ≥ 6 (50.0% vs. 20.0%, *p* = 0.015), INR ≥ 7 (43.3% vs. 16.7%, *p* = 0.024), INR ≥ 8 (40.0% vs. 13.3%, *p* = 0.020), INR ≥ 9 (33.3% vs. 6.7%, *p* = 0.010), and INR ≥ 10 (33.3% vs. 6.7%, *p* = 0.010).

### 3.4. Association Between Admission INR and In-Hospital Mortality

The relationship between admission INR and in-hospital mortality was subsequently examined within each treatment group (Table 3).

Among patients managed without PCC, survival status did not significantly differ across any of the evaluated INR thresholds. No admission INR cutoff demonstrated a significant association with in-hospital mortality in the non-PCC cohort. In exploratory within-group analyses, nominal associations were observed at selected INR thresholds among patients treated with 4F-PCC. At an INR threshold of 3, patients with INR ≥ 3 were more frequently observed among non-survivors (92.3% vs. 47.1%, *p* = 0.017). Similarly, at a threshold of 8, patients with INR ≥ 8 were more frequently observed among non-survivors than survivors (61.5% vs. 23.5%, *p* = 0.035). No other evaluated thresholds showed nominal associations with mortality within the 4F-PCC group. Given the small subgroup sizes and multiple threshold comparisons, these findings should be considered exploratory and should not be interpreted as identifying prognostic INR cutoffs.

Figure 1 illustrates the distribution of pretreatment INR values according to treatment allocation and in-hospital outcome. In the non-PCC group, deaths were predominantly observed among patients with lower INR values. In contrast, non-survivors in the 4F-PCC group exhibited a broader distribution of INR values, extending into markedly elevated ranges.

Overall, the histogram and subgroup analyses suggest that higher admission INR was associated with 4F-PCC administration, whereas its relationship with mortality was inconsistent. Notably, INR thresholds failed to discriminate survivors from non-survivors in patients managed without PCC, whereas only selected high-INR categories were associated with mortality among those receiving PCC.

### 3.5. Multivariable Analysis of In-Hospital Mortality

A Firth-penalized logistic regression analysis was performed to evaluate the association between 4F-PCC administration and in-hospital mortality after adjustment for measured potential confounders, including baseline neurological severity. One patient with unavailable admission hematoma volume was excluded, resulting in 59 patients with 25 in-hospital deaths in the complete-case model. After adjustment for age, admission INR, admission hematoma volume, comorbidity count, and admission GCS, 4F-PCC administration was not independently associated with in-hospital mortality (aOR 1.08, 95% CI 0.07–14.73, *p* = 0.952). Admission GCS was independently associated with in-hospital mortality, with higher GCS scores associated with lower odds of death (aOR 0.37 per 1-point increase, 95% CI 0.10–0.62, *p* < 0.001). Age, admission INR, admission hematoma volume, and comorbidity count were not independently associated with mortality in the revised model (Table 4).

## 4. Discussion

This multicenter cohort study evaluated the relationship between admission INR, 4F-PCC administration, and clinical outcomes in geriatric patients with non-traumatic WA-ICH. Several important findings emerged. First, higher admission INR values were associated with 4F-PCC administration, indicating that INR was related to treatment allocation in this cohort. Second, despite substantial baseline differences in anticoagulation intensity, the crude proportions of in-hospital mortality were similar between the treatment groups. This observation should not be interpreted as evidence of treatment equivalence or as evidence for or against a mortality benefit of 4F-PCC. Because treatment allocation was non-randomized and patients receiving 4F-PCC had higher baseline PT, aPTT, and INR values, the crude mortality comparison is particularly susceptible to confounding by indication. It should also be emphasized that the comparator represented a heterogeneous non-PCC management group rather than a uniform alternative reversal strategy; therefore, the between-group comparison should not be interpreted as a direct comparison of 4F-PCC with any single alternative reversal therapy. Third, admission INR was not consistently associated with mortality. Fourth, admission GCS was substantially lower among non-survivors and remained independently associated with in-hospital mortality after adjustment for measured clinical and radiological covariates. In contrast, 4F-PCC administration and admission INR were not independently associated with mortality in the revised Firth regression model. Collectively, these findings suggest that admission INR should not be interpreted in isolation when considering reversal therapy in geriatric WA-ICH and support consideration of a broader clinical and radiological assessment. However, given the retrospective design, small sample size, baseline differences in anticoagulation intensity, and potential residual confounding, these findings should be considered hypothesis-generating and do not establish that INR-based PCC selection is inadequate.

Current international guidelines recommend rapid reversal of VKA-associated ICH with 4F-PCC and intravenous vitamin K, emphasizing the need to minimize ongoing bleeding and prevent hematoma expansion [10,11,12,13]. However, these recommendations primarily address the urgency of reversal and the superiority of PCC over FFP in correcting coagulopathy rather than defining precise INR thresholds for treatment selection. In daily clinical practice, INR frequently functions not only as a dosing parameter but also as a determinant of whether PCC is administered. In our cohort, patients with progressively higher INR values were more likely to receive 4F-PCC, indicating an association between admission INR and treatment allocation.

Nevertheless, the prognostic significance of INR appears less straightforward. Although admission INR influenced treatment allocation, its relationship with in-hospital mortality was inconsistent. Notably, no INR threshold was associated with mortality in the non-PCC group, whereas exploratory analyses within the PCC-treated cohort showed nominal associations at selected INR thresholds. Given the small subgroup sizes and multiple comparisons, these observations should be considered hypothesis-generating and do not establish specific prognostic INR cutoffs. These findings suggest that anticoagulation intensity alone does not fully capture the complex determinants of outcome following WA-ICH. Clinical prognosis is likely influenced by a broader constellation of factors, including hematoma characteristics, neurological status, frailty, and comorbidity burden. In the present cohort, admission GCS did not differ significantly between the 4F-PCC and non-PCC groups but was markedly lower among non-survivors and remained independently associated with in-hospital mortality in the adjusted analysis. This finding highlights the prognostic relevance of baseline neurological severity beyond laboratory anticoagulation intensity. However, the present analysis was not designed to compare the prognostic performance of GCS and INR or to establish GCS as a treatment-selection criterion for anticoagulation reversal.

The management of patients with lower INR values represents a particularly important clinical challenge. Evidence supporting 4F-PCC administration is strongest in patients with INR ≥ 2, whereas individuals with lower INR levels have been underrepresented in randomized and observational studies [13]. Accordingly, current guideline recommendations are less definitive for patients with INR values below conventional treatment thresholds. Furthermore, the summary of product characteristics for the 4F-PCC formulation available in Turkey does not provide a clear dosing strategy for patients with bleeding and INR values below 2.2, potentially contributing to therapeutic uncertainty in clinical practice [7]. In our cohort, PCC administration was uncommon among patients with INR < 2, yet mortality remained substantial within this subgroup. These findings raise the possibility that clinically significant WA-ICH may occur even in the presence of relatively modest INR elevation and highlight the limitations of relying exclusively on INR-defined treatment thresholds.

In our cohort, post-treatment INR values were comparable between the groups, while unadjusted in-hospital mortality also did not differ significantly. However, these parallel findings should not be interpreted as evidence that 4F-PCC or INR correction lacks a survival benefit, because treatment allocation was non-random and patients receiving 4F-PCC had greater baseline anticoagulation intensity. Previous systematic reviews have consistently demonstrated the superiority of PCC over FFP in achieving faster INR normalization; however, evidence regarding mortality reduction remains heterogeneous [23]. In contrast, Kuramatsu et al. [24] reported that achieving rapid INR reversal to below 1.3 was associated with reduced hematoma expansion and lower mortality. The discrepancy between laboratory efficacy and clinical outcomes likely reflects the multifactorial nature of WA-ICH, where the timing of treatment, neurological injury severity, and baseline patient characteristics may produce effects that are at least as important as the degree of anticoagulation reversal itself. Therefore, although rapid correction remains a critical therapeutic goal, clinicians should avoid equating INR normalization with prognostic improvement.

Our findings showed that higher admission INR was associated with 4F-PCC administration, whereas its association with in-hospital mortality was inconsistent. This divergence suggests that outcomes in WA-ICH may be influenced by factors not captured by admission INR alone. One such factor highlighted in previous studies is the timing of anticoagulation reversal. Steiner et al. [25] demonstrated that PCC achieves more rapid reversal than FFP and may reduce hematoma progression through earlier restoration of coagulation function. Similarly, Sheth et al. [20], in a large multicenter analysis involving 9492 patients, reported significantly lower mortality among patients who received reversal treatment within 60 min of arrival at the hospital. Treatment timing and time to INR correction were not available in our cohort and therefore could not be examined directly; however, these studies may help contextualize the limited and inconsistent association between admission INR and clinical outcomes observed in our cohort.

An important strength of this study is our focus on a geriatric population, which remains underrepresented in the literature despite experiencing the greatest burden of WA-ICH morbidity and mortality. Older adults have unique physiological characteristics, including increased vascular fragility, diminished physiological reserve, multimorbidity, and polypharmacy, that may modify both the effectiveness and risks of reversal therapy.

An additional clinically relevant finding was the low and inconsistent use of concomitant vitamin K. Although contemporary guidelines recommend intravenous vitamin K alongside 4F-PCC to achieve sustained reversal of VKA anticoagulation, vitamin K was administered to only 40.0% of patients in the 4F-PCC group and 23.3% of those in the non-PCC group. Post-treatment INR values also remained above recommended targets, with values of 1.7 in the 4F-PCC group and 1.6 in the non-PCC group. Inconsistent vitamin K co-administration may have contributed to incomplete or less durable anticoagulation reversal and represents an important source of treatment heterogeneity that could have influenced clinical outcomes. Accordingly, the observed mortality patterns cannot be attributed to PCC administration alone. Future studies should evaluate standardized reversal protocols incorporating 4F-PCC with concomitant intravenous vitamin K and predefined INR reassessment.

Our findings should be interpreted in light of several limitations. First, the retrospective observational design introduces the potential for selection bias and unmeasured confounding. Second, treatment allocation was non-randomized, and patients receiving 4F-PCC had greater baseline anticoagulation intensity, including higher INR, PT, and aPTT values. This introduces confounding by indication and limits causal or comparative-effectiveness interpretation of the between-group mortality findings. In addition, residual confounding by unmeasured prognostic factors, particularly neurological severity and frailty, cannot be excluded. Moreover, vitamin K co-administration was neither universal nor standardized between treatment strategies, introducing further treatment heterogeneity and potentially influencing both the durability of INR reversal and clinical outcomes. Furthermore, patient-level 4F-PCC dose data were not consistently available, precluding assessment of dose–response relationships and introducing an additional source of treatment heterogeneity and residual confounding. Third, we were unable to evaluate important mechanistic variables, including door-to-treatment time and time-to-INR correction. In addition, the timing of follow-up CT imaging was not standardized across participating centers, which may have influenced the assessment of hematoma progression and limits the interpretation of between-group differences in radiological change. Fourth, the relatively small sample size reduced statistical power to detect clinically meaningful differences in mortality and resulted in sparse cell counts in several threshold-based subgroup analyses. Because multiple INR cutoffs were explored without adjustment for multiple testing, these analyses are susceptible to both type I and type II errors and should be interpreted as exploratory and hypothesis-generating rather than as evidence supporting specific INR thresholds. Although admission GCS could be retrieved for all included patients and was incorporated into the revised multivariable analysis, other potentially important measures of neurological and baseline clinical status, including NIHSS, pre-ICH functional status, and standardized frailty measures, were not consistently available across participating centers. Residual confounding related to these unmeasured factors therefore remains possible. In addition, although GCS captures an important dimension of neurological severity, it does not fully characterize focal neurological deficits or overall ICH severity.

## 5. Conclusions

Higher admission INR was associated with 4F-PCC administration, whereas admission INR and 4F-PCC administration were not independently associated with in-hospital mortality in the revised exploratory model. In contrast, admission GCS was independently associated with mortality, highlighting the prognostic relevance of baseline neurological severity in geriatric WA-ICH. These findings suggest that admission INR should not be interpreted in isolation when considering reversal therapy and that clinical and radiological context should also be taken into account. However, given the retrospective design, small sample size, non-randomized treatment allocation, baseline differences in anticoagulation intensity, heterogeneous management strategies, and potential residual confounding, these findings are hypothesis-generating and do not establish that INR-based PCC selection is inadequate or determine the comparative effectiveness of 4F-PCC.

Future studies should prospectively evaluate integrated approaches incorporating laboratory, neurological, and radiological variables to determine whether they improve treatment selection and prognostic assessment in geriatric WA-ICH.

## Figures and Tables

**Figure 1:**
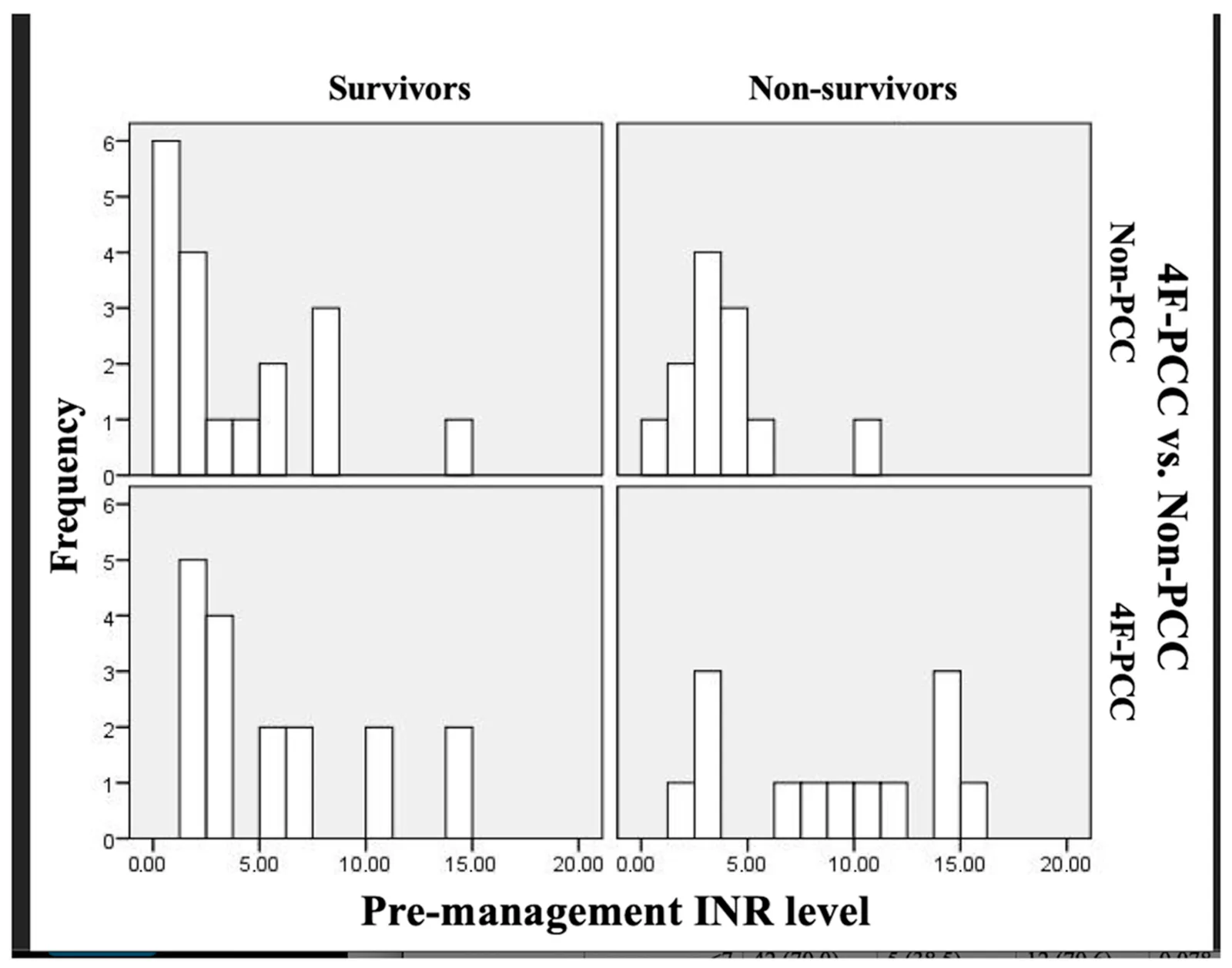
Distribution of pretreatment INR values according to treatment group (4F-PCC vs. non-PCC) and in-hospital outcome (survivors vs. non-survivors). The histogram illustrates differences in INR distributions between treatment groups and provides a visual representation of the relationship between admission anticoagulation intensity and mortality. INR, international normalized ratio; 4F-PCC, four-factor prothrombin complex concentrate.

**Table 1:** Comparison of baseline characteristics, hemorrhage features, reversal strategies, and in-hospital outcomes between patients treated with and without 4F-PCC.

	4F-PCC (*n* = 30)	Non-PCC (*n* = 30)	*p*-Value
**Sex, *n* (%)**			
Female	18 (60.0)	18 (60.0)	1.000
Male	12 (40.0)	12 (40.0)	
**Age in years, mean ± SD**	77.3 ± 8.5	75.5 ± 13.2	0.524
**Comorbidities, *n* (%)**			
Atrial fibrillation	13 (43.3)	18 (60.0)	0.196
Hypertension	22 (73.3)	22 (73.3)	1.000
Diabetes mellitus	8 (26.7)	9 (30.0)	0.774
Cardiovascular diseases	19 (63.3)	13 (43.3)	0.121
**Vital signs, mean ± SD**			
Systolic blood pressure, mmHg	139.1 ± 50.0	132.5 ± 44.0	0.750
Diastolic blood pressure, mmHg	80.0 ± 20.0	80.0 ± 18.2	0.688
Heart rate, bpm	88.5 ± 23.0	85.0 ± 21.0	0.605
**Admission GCS, median [IQR]**	12.5 [7.25–15]	13 [6–15]	0.880
**Coagulation parameters, median [IQR]**			
PT, seconds	51.95 [26.60–135.80]	31.00 [15.30–51.25]	0.018
aPTT, seconds	55.35 [37.65–74.73]	42.30 [30.40–53.63]	0.016
Pre-management INR level	5.90 [2.90–11.00]	3.15 [1.33–4.98]	0.005
Post-management INR level	1.70 [1.40–1.90]	1.65 [1.28–2.13]	0.476
Absolute INR reduction	4.30 [1.00–9.10]	2.00 [0.33–4.25]	0.104
**WA-ICH**			
Subarachnoid hemorrhage, *n* (%)	7 (23.3)	7 (23.3)	1.000
Intraventricular hemorrhage, *n* (%)	6 (20.0)	4 (13.3)	0.488
Admission intracerebral hemorrhage volume, mL, mean ± SD	19.7 ± 35.7	13.1 ± 34.2	0.306
Hematoma volume change on follow-up CT, %, mean ± SD	29.3 ± 166.7	56.5 ± 74.8	0.116
**Emergency department management, *n* (%)**			
Intubation in ED	15 (50.0)	10 (33.3)	0.190
FFP	3 (10.0)	5 (16.7)	0.706
Vitamin K	12 (40.0)	7 (23.3)	0.165
**Clinical disposition/outcome, *n* (%)**			0.952
Ward admission	9 (30.0)	9 (30.0)	
ICU admission	8 (26.7)	9 (30.0)	
In-hospital mortality	13 (43.3)	12 (40.0)	

**Note:** Data are presented as number of patients (*n*), percentage (%), mean ± standard deviation (SD), and median with interquartile range [IQR]. Between-group comparisons were performed using the independent-samples *t*-test or Mann–Whitney U test for continuous variables according to distribution, and the chi-square test or Fisher’s exact test for categorical variables, as appropriate. Absolute INR reduction was calculated as pre-treatment INR minus post-management INR. Hematoma volume was calculated using the ABC/2 method for measurable intraparenchymal hemorrhages. **Abbreviations:** 4F-PCC, Four-factor prothrombin complex concentrate; PT, prothrombin time; aPTT, activated partial thromboplastin time; INR, international normalized ratio; WA-ICH, Warfarin-associated intracranial hemorrhage; CT, computed tomography; FFP, fresh frozen plasma; ICU, intensive care unit.

**Table 2:** Treatment allocation according to admission INR thresholds in patients with WA-ICH.

Admission INR Threshold	Total (*n* = 60) *n* (%)	4F-PCC (*n* = 30) *n* (%)	Non-PCC (*n* = 30) *n* (%)	*p*-Value
**<2**	12 (20)	1 (3.3)	11 (36.7)	0.001
**≥2**	48 (80)	29 (96.7)	19 (63.3)	
**<3**	25 (41.7)	10 (33.3)	15 (50.0)	0.190
**≥3**	35 (58.3)	20 (66.7)	15 (50.0)	
**<4**	33 (55.0)	13 (43.3)	20 (66.7)	0.069
**≥4**	27 (45.0)	17 (56.7)	10 (33.3)	
**<5**	35 (58.3)	13 (43.3)	22 (73.3)	0.018
**≥5**	25 (41.7)	17 (56.7)	8 (26.7)	
**<6**	39 (65.0)	15 (50.0)	24 (80.0)	0.015
**≥6**	21 (35.0)	15 (50.0)	6 (20.0)	
**<7**	42 (70.0)	17 (56.7)	25 (83.3)	0.024
**≥7**	18 (30.0)	13 (43.3)	5 (16.7)	
**<8**	44 (73.3)	18 (60.0)	26 (86.7)	0.020
**≥8**	16 (26.7)	12 (40.0)	4 (13.3)	
**<9**	48 (80.0)	20 (66.7)	28 (93.3)	0.010
**≥9**	12 (20.0)	10 (33.3)	2 (6.7)	
**<10**	48 (80.0)	20 (66.7)	28 (93.3)	0.010
**≥10**	12 (20.0)	10 (33.3)	2 (6.7)	
**<12**	52 (86.7)	23 (76.7)	29 (96.7)	0.052
**≥12**	8 (13.3)	7 (23.3)	1 (3.3)	
**<14**	53 (88.3)	24 (80.0)	29 (96.7)	0.103
**≥14**	7 (11.7)	6 (20.0)	1 (3.3)	

**Note:** Data are presented as number of patients (*n*) and percentage (%). Percentages in the total column were calculated using the entire cohort as the denominator (*n* = 60), whereas percentages in the treatment columns were calculated using the corresponding treatment group as the denominator (*n* = 30 for each group). *p*-values represent between-group comparisons of the distribution below, versus, at, or above each INR threshold. The chi-square test or Fisher’s exact test was used, as appropriate. These threshold-based comparisons were exploratory and were not adjusted for multiple testing. Owing to the small sample size and sparse cell counts at several thresholds, the reported *p*-values should be interpreted cautiously and should not be considered evidence of validated INR cutoffs. **Abbreviations:** WA-ICH, Warfarin-associated intracranial hemorrhage; 4F-PCC, four-factor prothrombin complex concentrate; INR, international normalized ratio.

**Table 3:** Relationship between admission INR thresholds and in-hospital mortality in patients treated, or not, with 4F-PCC.

	4F-PCC (*n* = 30) *n* (%)			Non-PCC (*n* = 30) *n* (%)		
Admission INR Threshold	Non-Survivors (*n* = 13)	Survivors (*n* = 17)	*p*-Value	Non-Survivors (*n* = 12)	Survivors (*n* = 18)	*p*-Value
**<2**	0 (0.0)	1 (5.9)	1.000	3 (25.0)	8 (44.4)	0.442
**≥2**	13 (100.0)	16 (94.1)		9 (75.0)	10 (55.6)	
**<3**	1 (7.7)	9 (52.9)	0.017	4 (33.3)	11 (61.1)	0.136
**≥3**	12 (92.3)	8 (47.1)		8 (66.7)	7 (38.9)	
**<4**	4 (30.8)	9 (52.9)	0.225	9 (75.0)	11 (61.1)	0.694
**≥4**	9 (69.2)	8 (47.1)		3 (25.0)	7 (38.9)	
**<5**	4 (30.8)	9 (52.9)	0.225	10 (83.3)	12 (66.7)	0.419
**≥5**	9 (69.2)	8 (47.1)		2 (16.7)	6 (33.3)	
**<6**	4 (30.8)	11 (64.7)	0.065	11 (91.7)	13 (72.2)	0.358
**≥6**	9 (69.2)	6 (35.3)		1 (8.3)	5 (27.8)	
**<7**	5 (38.5)	12 (70.6)	0.078	11 (91.7)	14 (77.8)	0.626
**≥7**	8 (61.5)	5 (29.4)		1 (8.3)	4 (22.2)	
**<8**	5 (38.5)	13 (76.5)	0.035	11 (91.7)	15 (83.3)	0.632
**≥8**	8 (61.5)	4 (23.5)		1 (8.3)	3 (16.7)	
**<9**	7 (53.8)	13 (76.5)	0.193	11 (91.7)	17 (94.4)	1.000
**≥9**	6 (46.2)	4 (23.5)		1 (8.3)	1 (5.6)	
**<10**	7 (53.8)	13 (76.5)	0.193	11 (91.7)	17 (94.4)	1.000
**≥10**	6 (46.2)	4 (23.5)		1 (8.3)	1 (5.6)	
**<12**	8 (61.5)	15 (88.2)	0.190	12 (100.0)	17 (94.4)	1.000
**≥12**	5 (38.5)	2 (11.8)		0 (0.0)	1 (5.6)	
**<14**	9 (69.2)	15 (88.2)	0.360	12 (100.0)	17 (94.4)	1.000
**≥14**	4 (30.8)	2 (11.8)		0 (0.0)	1 (5.6)	

**Note:** Data are presented as *n* (%). Comparisons were performed within each treatment group according to survival status. Fisher’s exact test or the chi-square test was used, as appropriate. These threshold-based comparisons were exploratory and were not adjusted for multiple testing. Owing to the small sample size and sparse cell counts at several thresholds, the reported *p*-values should be interpreted cautiously and should not be considered evidence of validated INR cutoffs. **Abbreviations:** INR, international normalized ratio; 4F-PCC, four-factor prothrombin complex concentrate.

**Table 4:** Firth-penalized logistic regression analysis of factors associated with in-hospital mortality.

Variable	aOR	95% CI	*p*-Value
4F-PCC administration	1.08	0.07–14.73	0.952
Age, per 1-year increase	1.03	0.88–1.23	0.680
Admission INR, per 1-unit increase	0.90	0.54–1.17	0.480
Admission hematoma volume, per 10-mL increase	0.75	0.37–1.24	0.389
Comorbidity count, per 1-condition increase	1.24	0.31–8.11	0.694
Admission GCS, per 1-point increase	0.37	0.10–0.62	<0.001

**Note:** Comorbidity count represents the number of atrial fibrillation, hypertension, diabetes mellitus, and cardiovascular disease diagnoses recorded for each patient (range 0–4). The model included 59 complete cases and 25 in-hospital deaths; one patient was excluded because admission hematoma volume was unavailable. Odds ratios and 95% confidence intervals were estimated using Firth-penalized logistic regression. *p*-values were obtained using penalized likelihood-ratio tests. **Abbreviations:** aOR, adjusted odds ratio; CI, confidence interval; 4F-PCC, four-factor prothrombin complex concentrate; INR, international normalized ratio; GCS, Glasgow Coma Scale.

## Data Availability

The data presented in this study are available on request from the corresponding author due to privacy and ethical restrictions regarding patient confidentiality.

## Abbreviations

4F-PCC	Four-factor prothrombin complex concentrate
ACC	American College of Cardiology
AHA	American Heart Association
ASA	American Stroke Association
aPTT	Activated partial thromboplastin time
CT	Computed tomography
ED	Emergency department
FFP	Fresh frozen plasma
ICH	Intracranial hemorrhage
INR	International normalized ratio
PT	Prothrombin time
VKA	Vitamin K antagonist
WA-ICH	Warfarin-associated intracranial hemorrhage
